# Predictors and outcomes of respiratory bacterial coinfections in patients with COVID‐19 admitted to hospital: An observational prospective study

**DOI:** 10.1111/resp.14372

**Published:** 2022-09-16

**Authors:** Giusy Tiseo, Lorenzo Roberto Suardi, Lisa Giusti, Arianna Forniti, Claudio Caroselli, Valentina Galfo, Sara Occhineri, Alessandro Leonildi, Giovanna Moscato, Francesco Menichetti, Marco Falcone

**Affiliations:** ^1^ Infectious Diseases Unit, Department of Clinical and Experimental Medicine Azienda Ospedaliera Universitaria Pisana, University of Pisa Pisa Italy; ^2^ Microbiology Unit Azienda Ospedaliera Universitaria Pisana Pisa Italy; ^3^ Seroimmunology Unit Pisa University Hospital Pisa Italy

**Keywords:** bacterial infection, coinfections, coronavirus disease, COVID‐19, procalcitonin, SARS‐CoV‐2, Streptococcus pneumoniae

## Abstract

No abstract available.

## Graphical abstract

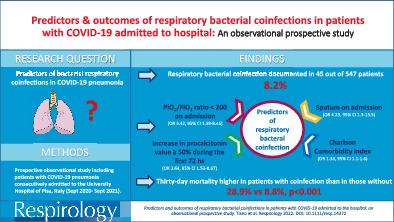

To the Editors:

Bacterial coinfections are common complications of viral respiratory infections. In patients with influenza, bacterial coinfections range from 2% to 65%.^1^ In patients with coronavirus disease 2019 (COVID‐19) coinfections are less common (<10%).^2^, ^3^ The public health organizations warned against the indiscriminate use of antibiotics in COVID‐19 to avoid selection of antimicrobial resistance and reduce the risk of bacterial superinfections.^4^ Unlike coinfections, superinfections occurring during hospital course are common and associated with inappropriate antibiotic use on admission.^5^, ^6^ Unfortunately, antibiotic prescriptions in COVID‐19 patients remain unacceptably high.^7^

We performed a prospective, observational study to identify predictors of respiratory coinfections in COVID‐19. Patients with COVID‐19 pneumonia consecutively admitted to the University Hospital of Pisa, Italy (September 2020–September 2021) were included. All patients were prospectively followed‐up from Emergency Department (ED) admission to hospital discharge or death. Both microbiological and laboratory exams were performed according to standards of practice and attending physicians judgement. Microbiological examinations were performed on sputum, urine and blood during the first 24 h after admission. Results of tracheobronchial aspirates, and bronchoalveolar lavage fluid (BAL), when performed, were considered as well as serologic tests for *Chlamydophila pneumoniae, Legionella pneumophila* or *Mycoplasma pneumoniae*. The aetiology was considered definite if 1 of the following criteria was met: positive urinary antigen for *Legionella pneumophila* or *Streptococcus pneumoniae*; bacterial yield in cultures from valid sputum (>25 polymorphonuclear cells and 10 epithelial cells per high‐power field) of 10^6^ CFU/mL, from tracheobronchial aspirates of 10^5^ CFU/mL, from BAL fluid of 10^4^ CFU/mL or from protected specimen brush cultures of 10^3^ CFU/mL; or occurrence of seroconversion (a four‐fold increase in IgG titers for *C. pneumoniae* [1:512] or an increase in IgM titers for *C. pneumoniae* [1:32] or *M. pneumoniae* [any titer]).^8^ Charlson Comorbidity index was calculated for each patient.^9^ Respiratory coinfections were defined as infections diagnosed within the first 24 h from hospital admission.^2^ Superinfections were infections diagnosed more than 48 h after admission.^2^ The primary objective was to identify factors independently associated with bacterial respiratory coinfections. The secondary objective was to explore the impact of coinfections on a composite endpoint of death or need for invasive mechanical ventilation within 30 days from admission.

Multivariable regression analyses were performed to identify predictors of coinfection and predictors of the composite endpoint, using a forward stepwise procedure, and entering all variables with univariate *p*  <  0.05 and those of clinical relevance. For the composite endpoint, the time at risk was defined as the days between hospital admission and the occurrence of endotracheal intubation or death. Statistical significance was established at *p*  <  0.05. The analysis was performed using a commercially available statistical software package (SPSS 27.0; IBM, Armonk, NY).

Overall, 547 patients with COVID‐19 were included. Median age was 68 ys (IQRs 57‐80). Broad‐spectrum antibiotics were started in 270 (49.4%) patients within 48 h from hospital admission. A total of 144 (26.3%) patients already received antibiotics at home. All patients underwent urinary antigen and serological tests. The 30‐day mortality rate was 10.4%. A total of 45 (8.2%) patients had a respiratory coinfection: 36/45 (80%) by *Streptococcus pneumoniae*, 6/45 (13.3%) by *Mycoplasma pneumoniae*, 1/45 (2.2%) by *Corynebacterium* spp, 1/45 (2.2%) by *Staphylococcus aureus*, and 1/45 (2.2%) by *Klebsiella pneumoniae*. The positive specimens were: 32 positive urinary antigens (32 *S. pneumoniae*), 6 serological tests (6 *M. pneumoniae*), 5 positive sputum (4 *S. pneumoniae*, 1 *K. pneumoniae*) and 2 BAL (1 *S. aureus and* 1 *C. striatum*). All specimens were collected before the start of empiric antibiotic therapy at the ED. All patients underwent CT scan on admission and 40/45 (88.9%) had parenchymal consolidations. Among patients who already received antibiotics at home, 8/144 (5.5%) had a coinfection.

Conversely, 145 (26.5%) patients developed a bacterial superinfection during hospital course: 84 urinary‐tract infections, 45 bloodstream infections, 14 low‐respiratory tract infections, 2 skin infections.

Compared to subjects with no respiratory coinfection, coinfected patients were more frequently affected by chronic obstructive pulmonary disease (20% vs. 10.2%, *p* = 0.04), cerebrovascular disease (22.2% vs. 11.8%, *p* = 0.04), chronic kidney disease (24.4% vs. 7%, p < 0.001), solid cancer (20% vs. 9%, *p* = 0.02) and had a higher Charlson Comorbidity Index. They had more frequently sputum (11.1% vs. 2.8%, *p* = 0.003) and PiO_2_/FiO_2_ ratio <200 (17.8% vs. 5.4%, *p* = 0.001), both on admission to the ED. On laboratory exams, the following parameter alterations were more common in patients with coinfections than in those without: WBC > 10,000/mcl (37.8% vs. 22.9%, *p* = 0.03), and increase of the procalcitonin (PCT) value more than 50% during the first 72 h from admission (20% vs. 5.8%, *p* < 0.001). PCT values were available for 494 (90.3%) patients. Thirty‐day mortality rate was higher in coinfected patients than in subjects without coinfection (28.9% vs. 8.8%, *p* < 0.001). On multivariable analysis, PiO_2_/FiO_2_ ratio <200, presence of sputum on admission, increase in the PCT value ≥50% within the first 72 h and Charlson Comorbidity Index were factors independently associated with respiratory coinfection (Table 1).

**TABLE 1:** Multivariable analysis of factors independently associated with respiratory bacterial coinfections and with the composite endpoint (need for mechanical ventilation or death) in hospitalized COVID‐19 patients

Factors associated with respiratory coinfections			Factors associated with the composite endpoint		
Multivariable logistic regression analysis			Multivariable Cox regression analysis		
Variable^a^	OR (95% CI)	*p* value	Variable^b^	HR (95% CI)	*p* value
PiO_2_/FiO_2_ ratio <200 on admission	3.42 (1.39–8.45)	0.008	Respiratory coinfection	2.9 (1.17–7.06)	0.021
Sputum on admission	4.23 (1.3–13.5)	0.015	Age	1.03 (1.01–1.06)	0.011
PCT increase ≥50% during the first 72 h^c^	3.64 (1.53–8.67)	0.003	Ferritin value on admission	1 (1.0–1.1)	<0.001
Charlson Comorbidity Index	1.33 (1.1–1.6)	0.003	Charlson Comorbidity Index	1.29 (1.1–1.52)	0.002

^a^ Variables included and not retained in the model: age, cerebrovascular disease, COPD, chronic kidney disease (CKD), solid cancer, WBC >10,000/mcL, Lymphopenia (L < 800/mcL).

^b^ Variables included and not retained in the model: cardiovascular disease, hypertension, COPD, CKD, days from onset of symptoms, dyspnoea on admission, PiO_2_/FiO_2_ ratio < 200 on admission, C‐reactive protein and PCT values on admission, superinfection.

c PCT values were available for 494 (90.3%) patients. Charlson Comorbidity Index were calculated according to Reference 9.

A total of 71 (13%) patients met the composite endpoint. On Cox regression analysis, respiratory coinfections, Charlson Comorbidity Index, age and ferritin value on admission were predictors of death or endotracheal intubation (Table 1).

In this study, we found that respiratory coinfections occur in 8% of hospitalized patients with COVID‐19 and are associated with high risk of progression to severe COVID‐19. The proportion of coinfections changed from the beginning of the pandemic. The study by Garcia‐Vidal et al. conducted during the first wave reported a prevalence of 3.1%.^2^ A more recent observational study by Moreno‐Garcia et al. (February 2020–February 2021) reported that coinfections occurred in 9% of patients.^10^ Although in this study both respiratory coinfections and bacteraemia caused by *E. coli*, *S. aureus*, *P. aeruginosa* and *H. influenzae* were included, the results are similar to our findings. During the course of the pandemic, a wide use of steroids—frequently administered also before hospital admission—may have contributed to an increase in the proportion of bacterial coinfections. Moreover, since disease severity has decreased during time according to vaccination and spread of new variants (e.g., omicron), factors such as coinfections might more strongly contribute to disease severity causing hospital admission. The Spanish colleagues found that patients with higher PCT values had more risk to be affected by coinfections than those with lower levels.^10^ We found that the trend in PCT values (increase ≥50% in the first 72 h from admission) instead of the absolute value is associated with increased risk of coinfections, highlighting the need of PCT monitoring during the first 3 days from hospital admission.

Our findings are also in line with the study by Russell CD^7^. However, in this study *S. aureus* and *H. influenzae* were more frequently identified compared to our study. This may be due to different local epidemiology, more strict microbiological surveillance and differences in the patient population. In fact, Russell included a higher proportion of immunocompromized patients (29.3%) and chronic obstructive pulmonary disease (COPD; 28.7%)^7^ compared to our study (16.6% and 11%, respectively).

We found an association between coinfections and severe COVID‐19. This relationship may be dual. First, patients with coinfections may have an increased risk of disease progression; thus, identification of coinfected patients may allow physicians to select patients who need antibiotic therapy. Second, it is also possible that patients with more severe disease had a high risk of presenting with bacterial coinfections on admission. However, the indiscriminate use of antibiotics in all patients with severe COVID‐19 should be avoided and not encouraged because of the risk of multidrug‐resistant organisms selection. Thus, implementing the diagnosis of coinfections on ED and correlating microbiological data with radiological, clinical and laboratory features may guide the start of antibiotic therapy only in patients who really need antibiotics. The start of antibiotic therapy on admission should be guide by several factors, such as disease severity and increase in PCT values. Subjects with mild to moderate COVID‐19 who need oxygen therapy without an increase in the PCT levels during the first 3 days from hospital admission, empiric antibiotic therapy may be avoided.

Our study has some limitations: (i) it is an observational single‐centre study and the sample size is relatively low; (ii) the association between coinfections and poor outcome might be related to higher disease severity of patients with coinfections; (iii) we did not consider the impact of treatments on the outcome of patients, although in our hospital we used a standardized therapeutic approach based on an internal guide for the management of COVID‐19, updated on regularly basis.^11^

In conclusion, the identification of COVID‐19 patients with a respiratory coinfection on hospital admission is crucial to reduce inappropriate antibiotic use and to select those subjects at higher risk of mortality who may beneficiate from antibiotic therapy. Our findings may help clinicians to avoid inappropriate antibiotic therapy in patients with COVID‐19 admitted to the hospital.

## AUTHOR CONTRIBUTION

**Giusy Tiseo:** Conceptualization (lead); data curation (lead); formal analysis (lead); investigation (equal); methodology (lead); validation (lead); visualization (equal); writing – original draft (lead); writing – review and editing (lead). **Lorenzo Roberto Suardi:** Data curation (equal); investigation (equal); methodology (equal); validation (equal); visualization (equal); writing – review and editing (equal). **Lisa Giusti:** Data curation (equal); formal analysis (supporting); investigation (equal); methodology (equal); validation (equal); visualization (equal); writing – review and editing (equal). **Arianna Forniti:** Data curation (equal); methodology (equal); validation (equal); visualization (equal); writing – review and editing (equal). **Claudio Caroselli:** Data curation (equal); formal analysis (supporting); methodology (equal); validation (equal); visualization (equal); writing – review and editing (equal). **Valentina Galfo:** Data curation (equal); formal analysis (supporting); investigation (equal); methodology (equal); validation (equal); visualization (equal); writing – review and editing (equal). **Sara Occhineri:** Data curation (equal); formal analysis (supporting); methodology (equal); validation (equal); visualization (equal); writing – review and editing (equal). **Alessandro Leonildi:** Data curation (equal); formal analysis (supporting); methodology (equal); validation (equal); visualization (equal); writing – review and editing (equal). **Giovanna Moscato:** Data curation (equal); formal analysis (supporting); methodology (equal); validation (equal); visualization (equal); writing – review and editing (equal). **Francesco Menichetti:** Conceptualization (supporting); data curation (equal); formal analysis (supporting); investigation (equal); methodology (equal); validation (equal); visualization (equal); writing – review and editing (equal). **Marco Falcone:** Conceptualization (lead); data curation (lead); formal analysis (lead); investigation (equal); methodology (equal); supervision (lead); validation (lead); visualization (equal); writing – original draft (supporting); writing – review and editing (equal).

## CONFLICTS OF INTEREST

Giusy Tiseo received honoraria for educational activity from Shionogi. Marco Falcone received grants or speaker honoraria from MSD, Angelini, Shionogi, Pfizer, Menarini and Nordic Pharma. Francesco Menichetti has participated in advisory boards and/or received speaker honoraria from Angelini, Correvio, Merck Sharp & Dohme (MSD), Nordic Pharma, Pfizer, Astellas, Gilead, Bristol‐Myers Squibb (BMS), Janssen, ViiV, bioMérieux, Biotest, Becton Dickinson, Pfizer and Shionogi. The remaining authors have nothing to disclose.

## HUMAN ETHICS APPROVAL DECLARATION

This study was approved by the Omitato Etico Area Nord‐Ovest (CEAVNO, approval number 17681). Informed consent was received from all patients.

## Supporting information


**Visual Abstract** Predictors & outcomes of respiratory bacterial coinfections in patients with COVID‐19 admitted to hospital: An observational prospective study

## Data Availability

Data are available on request to the corresponding author.
