# Supplementary material for: Predictors and outcomes of respiratory bacterial coinfections in patients with COVID‐19 admitted to hospital: An observational prospective study
Source: Respirology. 2022 Sep 16:10.1111/resp.14372. Online ahead of print. doi: 10.1111/resp.14372 (PMC9538170; doi:10.1111/resp.14372)
Supplement: Supplementary file 1 — Visual Abstract Predictors & outcomes of respiratory bacterial coinfections in patients with COVID‐19 admitted to hospital: An observational prospective study [file RESP-9999-0-s001.pdf]

Predictors & outcomes of respiratory bacterial coinfections in patients with COVID-19 admitted to hospital: An observational prospective study

RESEARCH QUESTION

Predictors of bacterial respiratory coinfections in COVID-19 pneumonia

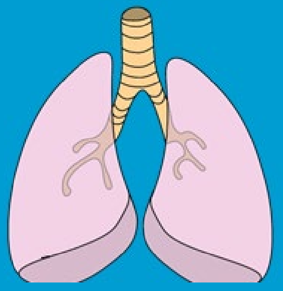

?

METHODS

Prospective observational study including patients with COVID-19 pneumonia consecutively admitted to the University Hospital of Pisa, Italy (Sept 2020- Sept 2021).

FINDINGS

Respiratory bacterial coinfection documented in 45 out of 547 patients

8.2%

Predictors of respiratory bacteral coinfection

PiO_2/FiO_2 ratio < 200 on admission
(OR 3.42, 95% CI 1.39-8.45)

Sputum on admission
(OR 4.23, 95% CI 1.3-13.5)

Increase in procalcitonin value ≥ 50% during the first 72 hs
(OR 3.64, 95% CI 1.53-8.67)

Charlson Comorbidity Index
(OR 1.33, 95% CI 1.1-1.6)

Thirty-day mortality higher in patients with coinfection than in those without

28.9% vs 8.8%, p<0.001

Respirology

Predictors and outcomes of respiratory bacterial coinfections in patients with COVID-19 admitted to the hospital: an observational prospective study. Tiseo et al. Respirology 2022: DOI: 10.1111/resp.14372
